# Eye movement characteristics and their correlation with cognitive function in cerebral small vessel disease

**DOI:** 10.3389/fneur.2026.1878897

**Published:** 2026-08-11

**Authors:** Fangming Wang, Xiaoling Zhang, Xiaotong Zhang, Litao Li

**Affiliations:** Department of Neurology, Hebei General Hospital, Shijiazhuang, Hebei, China

**Keywords:** cerebral small vessel disease, characteristics, cognitive impairment, eye movements, saccade

## Abstract

**Objective:**

This study aimed to investigate the characteristics of eye movements and their correlation with cognition in patients with cerebral small vessel disease (CSVD).

**Methods:**

This was a cross-sectional study enrolling a total of 120 patients with CSVD admitted to Hebei General Hospital from January 2022 to October 2023. The videonystagmography (VNG) was completed, and the Montreal Cognitive Assessment (MoCA) was used to evaluate the patients’ cognitive function. Patients were stratified into mild (0), moderate (1–2), and severe (3–4) groups based on a 4-point MRI score integrating white matter hyperintensities, lacunar infarcts, enlarged perivascular spaces, and cerebral microbleeds.

**Results:**

Decreased right saccade accuracy (OR = 0.923, 95% CI: 0.890 ~ 0.959, *p* < 0.001), decreased left saccade accuracy (OR = 0.938, 95% CI: 0.908 ~ 0.968, *p* < 0.001), prolonged right saccade latency (OR = 1.015, 95% CI: 1.008 ~ 1.023, *p* < 0.001), and prolonged left saccade latency (OR = 1.014, 95% CI: 1.007 ~ 1.022, p < 0.001) were independently associated with increased total CSVD burden. Decreased right saccade accuracy (OR = 0.924, 95% CI: 0.876 ~ 0.973, *p* = 0.004), decreased left saccade accuracy (OR = 0.944, 95% CI: 0.903 ~ 0.988, *p* = 0.012), decreased right peak velocity (OR = 0.988, 95% CI: 0.978 ~ 0.998, *p* = 0.030), decreased left peak velocity (OR = 0.986, 95% CI: 0.976 ~ 0.996, *p* = 0.007) and increased rightward slow-phase velocity values (OR = 4.668, 95% CI: 1.669 ~ 13.066, *p* = 0.003) were independently associated with cognitive impairment.

**Conclusion:**

Eye movement characteristics in CSVD patients were significantly correlated with the total MRI load and cognitive function. This provides an important reference value for the early identification and assessment of CSVD severity.

## Introduction

Cerebral small vessel disease (CSVD) is a major contributor to stroke and dementia (1). Its clinical manifestations are broad, encompassing gait disturbances, mood disorders, and urinary dysfunction (2). Cognitive impairment, particularly in executive function and processing speed, is the most prominent feature and places a significant burden on patients and society (3, 4). Early identification of CSVD is difficult because its onset and progression are often insidious. Neuroimaging markers such as white matter hyperintensities and lacunes are the diagnostic standard, but they reflect static structural damage rather than dynamic functional status (5). Conventional cognitive screening tools are also limited by educational and linguistic differences (6). An objective, non-invasive tool that assesses functional abnormalities in addition to structural damage would therefore be valuable for improving CSVD evaluation.

Oculomotor function relies on the precise integration of widely distributed cortical and subcortical networks (7), making it a sensitive barometer of overall brain health. VNG is used to record eye movements with a camera and analyze various eye movements. Compared with general imaging, VNGs are easier to perform, less invasive and better suited to the early assessment and monitoring of neurological disorders (8). Prior studies reported that oculomotor abnormalities correlate with cognitive decline in patients with CSVD (9), whereas impaired cortical–subcortical connectivity underlies oculomotor deficits (10). Voss et al. (11) analyzed oculomotor metrics from pro-saccade, anti-saccade, smooth pursuit and fixation via partial least squares regression, verifying that eye-tracking parameters act as markers of cognitive performance. Such oculomotor-cognition correlations are markedly stronger in neurodegenerative patients, owing to impaired shared frontostriatal and parietal circuits governing executive function, attention and processing speed alongside eye movement control (11). However, the relationship between abnormal eye movements and the severity of CSVD as well as cognitive impairment remains unclear.

Based on this, this study analyzed the oculomotor characteristics of different CSVD MRI manifestations, exploring the relationship between CSVD oculomotor characteristics and cognitive function. The aim was to provide a reference for the clinical recognition and assessment of CSVD.

## Methods

### Subjects

A total of 120 patients with cerebral small vessel disease (CSVD) who were hospitalized at Hebei Provincial People’s Hospital between January 2022 and October 2023 were selected for the study. Patients were screened according to the inclusion and exclusion criteria, and underwent VNG testing and refinement of the MoCA scale. The study protocol was approved by the Ethics Committee of Hebei Provincial People’s Hospital. The study was conducted in accordance with the Declaration of Helsinki, and written informed consent was obtained from all participants prior to enrollment.

Inclusion criteria: (1) age ≥50 years; (2) cranial MRI manifestations of one or more of WMH, lacunes, PVS, and CMB; (3) blood tests, MRI of the head, including diffusion weighted imaging (DWI), magnetic resonance angiography Magnetic resonance angiography (MRA), cranial T1-weighted image (T1WI), cranial T2-weighted image (T2WI), fluid attenuated inversion recovery (FLAIR), and magnetic susceptibility weighted imaging (SWI), and complete clinical, laboratory, and imaging data.

Exclusion criteria: (1) vestibular peripheral vertigo disease; (2) the presence of ocular diseases, acute cerebrovascular events or psychiatric disorders such as anxiety, etc.; (3) other diseases that cause eye movement disorders; (4) people with large cerebral infarcts (cranial MRI suggests that the infarcted area has a maximum diameter of >20 mm); (5) people with large vessel lesions, cardiogenic embolism, traumatic brain injury, intracranial infection, intracranial occupancy syndrome, hydrocephalus, or cerebral hemorrhage; (6) non-vascular source of cerebral white matter lesions; (7) presence of severe medical illness; (8) taking tranquilizers, sedatives, vestibular depressants, or alcoholic beverages within 48 h; (9) patients with incomplete clinical data.

#### Clinical data collection

Data on baseline demographics, risk factors (such as alcohol consumption level), medical history (including blood lipid levels, past history of diabetes, hypertension, and coronary heart disease), were collected. Baseline fasting venous blood samples were collected for laboratory analysis, encompassing a range of laboratory tests, including low-density lipoprotein (LDL; normal range: 2.1–3.1 mmol/L) and neutrophils (normal range: 0.40–0.75).

#### Scoring and grouping of CSVD total load

Cranial scans were performed using a 3.0 TeslaSingna HD superconducting MRI scanner manufactured by GE, USA, whose scanning sequences included T1WI, T2WI, FLAIR, DWI, and SWI. The images were evaluated by two neurologists, and in case of disagreement, a third neurologist of senior rank reviewed the images. The total CSVD burden score was computed by summing ratings of four predefined MRI markers (WMH, LI, PVS, CMB) following the grading system established by Staals et al. (12)^.^ One point was awarded for each criterion: (1) Fazekas score 3 for periventricular WMH or ≥ 2 for deep WMH; (2) ≥ 1 LI; (3) ≥ 1 CMB; (4) ≥ 11 PVS in the basal ganglia. Total scores ranged from 0 to 4. All imaging assessments were completed by two independent neuroradiologists blinded to clinical information to reduce evaluator bias. Patients were stratified into mild (0), moderate (1–2), and severe (3–4) groups according to their CSVD burden scores.

#### VNG examination

The VNG examination was performed using a Danish ICS Impulse System Scanner, which included a test for spontaneous nystagmus, a gaze test, a saccade test, an alternating cover test, and a vestibulo-ocular reflex test. Patients were seated with their upper body kept still and wearing video glasses. The system was then adjusted for correct pupil identification and tracking before the following steps were performed.

##### Steps for examining spontaneous nystagmus

The patient’s head was kept still. A light cover was placed in front of the patient’s eyes to prevent light stimulation. The patient was instructed to open their eyes and look straight ahead. The observation time was more than 20 s. It was recorded whether or not there was spontaneous nystagmus.

##### Steps of the gaze test

The patient’s head was kept still and their eyes were positioned at the same level as the center target on the screen. The distance to the front target was 1.2 m. The patient was instructed to gaze in different directions (central parallax position; 30° for each of the left/right positions; 25° for each of the upper/lower positions) and the observation time in each direction was more than 20 s. The slow-phase velocity (SPV) value was recorded.

##### Steps of the saccade test examination

The patient’s head remained motionless while the eye mask projected a laser beam. The patient had to quickly follow the irregularly moving laser dot to detect the accuracy, peak velocity and latency of the eye movement. Peak velocity is the maximum velocity reached by the eye jump in 18.75 ms. Accuracy is the ratio of saccade amplitude to laser dot amplitude. Latency is the time between the laser dot moving and the first eye movement exceeding 108°/s.

##### Steps of the alternating masking test

The patient’s head remained still, with their eyes fixed on the center target on the screen directly in front of them. The tester alternately covered the patient’s right and left eyes and recorded the eye movements on the computer.

##### Steps of the vestibulo-ocular reflex test

(1) Vestibulo-ocular reflex test (VVOR): The patient was asked to relax their neck while staring at a fixed target on the screen in front of them. The tester held the patient’s head and shook it horizontally at 0.5 Hz and 15°, recording eye movements and observing for saccadic waves. (2) Vestibulo-ocular reflex suppression (VORS): The patient was asked to relax their neck and stare at a moving laser point projected onto the screen. The tester held the patient’s head and shook it horizontally at 0.5 Hz and 15° to record eye movements and observe for saccadic waves.

#### Cognitive function assessment

Specialized evaluators used the MoCA to assess the cognitive function of enrolled patients. According to their level of education and their MoCA score, patients were considered to have cognitive impairment if they scored ≤13 points if they were illiterate, ≤19 points if they had 1–6 years of education, or ≤24 points if they had 7 or more years of education. One point was added to the total MoCA score for those with ≤12 years of education. These specific thresholds were established based on validation studies conducted within the Chinese population (13–17). Among the 120 enrolled patients, 39 did not complete the cognitive assessment due to logistical or compliance-related factors. To evaluate potential selection bias, baseline characteristics were compared between participants with complete cognitive data (*n* = 81) and those with missing data (*n* = 39). The detailed comparison of all baseline variables is provided in Supplementary Table 1.

### Statistical analysis

Statistical analysis was performed using SPSS 26.0 software. Measurement data were tested for normality, and those with normal distribution were expressed as $\bar{x}$ ± S, and comparisons between groups were tested using the ANOVA test; those with non-normal distribution were expressed as medians and quartiles [*M* (P25, P75)], and comparisons between groups were tested using the multiple sample rank sum test. Frequencies and percentages were used to represent categorical variables. Correlations between CSVD MRI total load and eye movement data were analyzed using spearman correlation analysis. The independent variables with differences in univariate analysis were included in multivariate ordered logistic regression or binary logistic regression models to analyze the correlation between total CSVD load or cognitive function and eye movement. To correct for multiple comparisons, *p*-values obtained from multiple group comparisons were adjusted using the Benjamini-Hochberg False Discovery Rate (FDR) method. *Post hoc* pairwise comparisons between groups were performed with Bonferroni correction. The value of eye movement characteristics in assessing the severity of CSVD and cognitive function were further analyzed by the Receiver operation characteristic (ROC) curve of the subjects, and the results were expressed as the area under the curve (AUC) of the ROC curve. The difference was considered statistically significant at *p* < 0.05.

## Results

### Oculomotor characteristics of CSVD

#### General information of CSVD patients

A total of 120 CSVD patients were included, with ages ranging from 50 to 81 years old and a median of 68 years old; with 69 males and 51 females. CSVD total load score: 0 score, 19 cases, 15.8%; 1 score, 32 cases, 26.7%; 2 score, 26 cases, 21.7%; 3 score, 31 cases, 25.8%; 4 score, 12 cases, 10%; 19 people in the mild CSVD total load group, 58 people in the moderate group, and 43 people in the severe group (see Table 1).

**Table 1:** General information comparison of different CSVD imaging total loads.

Variables	Total (*n* = 120)	Mild group (*n* = 19)	Moderate group (*n* = 58)	Severe group (*n* = 43)	*p*
Age, years [M (IQR)]	68 (13)	66 (10)	64 (15)	71 (9)	**0.002**
Sex					0.107
Female [*n* (%)]	51 (42.5)	11 (57.9)	25 (43.1)	15 (34.9)	
Male [*n* (%)]	69 (57.5)	8 (42.1)	33 (56.9)	28 (65.1)	
Smoking [*n* (%)]	40 (33.3)	5 (26.3)	17 (29.3)	18 (41.9)	0.150
Alcohol [*n* (%)]	24 (20.0)	2 (10.5)	11 (19.0)	11 (25.6)	0.172
Hypertension [*n* (%)]	82 (68.3)	8 (42.1)	37 (63.8)	37 (86.0)	**<0.001**
Diabetes [*n* (%)]	24 (20.0)	2 (10.5)	11 (19.0)	11 (25.6)	0.172
Coronary artery disease [*n* (%)]	17 (14.2)	3 (15.8)	10 (17.2)	4 (9.3)	0.334
Hyperlipidemia [*n* (%)]	24 (20.0)	3 (15.8)	8 (13.8)	13 (30.2)	0.070
LDL (mmol/l) [M (IQR)]	2.67 (1.17)	3.05 (1.12)	2.50 (1.22)	2.47 (1.10)	0.597
Neutrophils (%) [M (IQR)]	63.3 (13.6)	60.0 (16.9)	62.2 (14.2)	63.7 (11.3)	0.659

Bold values indicate statistically significant differences (*p* < 0.05).

There was a difference in age between the three groups of patients (*p* < 0.01), and the age of the CSVD severe group was greater than that of the CSVD moderate group (*p* < 0.05); there was a difference in the history of suffering from hypertension between the three groups (*p* < 0.05); and there was no statistically significant difference in gender, history of alcohol consumption, diabetes mellitus, coronary artery disease, hyperlipidaemia, LDL, and neutrophils between the three groups (*p* > 0.05) (see Table 1).

#### Eye movement characteristics of CSVD patients

In the three groups, there were differences in right saccade accuracy, left saccade accuracy, right peak velocity, left peak velocity, right saccade latency, and left saccade latency in the saccade test (*p* < 0.05). *Post hoc* pairwise comparisons demonstrated that patients in the mild and moderate groups had higher bilateral saccade accuracy and shorter latency relative to the severe group (*p* < 0.05). In addition, the moderate group exhibited significantly greater peak velocity than the severe group (*p* < 0.05). There were differences in the angle of obliquity in the alternating masking experiment and the downward SPV values in the gaze experiment (*p* < 0.05). There were no differences in the centered SPV values, leftward SPV values, rightward SPV values, and upward SPV values in the gaze experiment (*p* > 0.05). No spontaneous nystagmus and vestibular reflex saccading waves were detected in any of the three groups (see Table 2).

**Table 2:** Comparison of eye movement data of different CSVD imaging total loads.

Variables	Total (*n* = 120)	Mild group (*n* = 19)	Moderate group (*n* = 58)	Severe group (*n* = 43)	*p*	FDR-adjusted *p*-value
Right saccade accuracy (%)	73.44 ± 11.42	78.84 ± 9.99^c^	76.90 ± 8.85^c^	66.40 ± 11.8^ab^	**<0.001**	**0.0012**
Left saccade accuracy (%)	76.37 ± 13.56	84.74 ± 14.02^c^	79.38 ± 10.83^c^	68.60 ± 13.11^ab^	**<0.001**	**0.0006**
Right peak velocity (°/s)	257.57 ± 59.56	267.11 ± 54.50	271.57 ± 63.20^c^	234.47 ± 49.04^b^	**0.005**	**0.0086**
Left peak velocity (°/s)	278.51 ± 62.34	293.16 ± 60.28	294.03 ± 59.49^c^	251.09 ± 58.76^b^	**0.001**	**0.0024**
Right saccade latency (ms)	245.48 ± 55.03	206.00 ± 32.70^c^	235.50 ± 55.81^c^	276.40 ± 45.55^ab^	**<0.001**	**0.0004**
Left saccade latency (ms)	259.26 ± 56.50	217.16 ± 28.3^c^	251.57 ± 44.15^c^	288.23 ± 65.68^ab^	**<0.001**	**0.0003**
Obliquity angle (°)	0.9 (0.9)	0.5 (0.7)	1.1 (0.8)	0.8 (0.9)	**0.002**	**0.0040**
Centered SPV (°/s)	0.6 (0.6)	0.7 (1.0)	0.6 (0.5)	0.7 (0.7)	0.834	0.8340
Leftward SPV (°/s)	0.6 (0.7)	0.4 (0.7)	0.6 (0.5)	0.7 (0.8)	0.117	0.1404
Rightward SPV (°/s)	0.7 (0.7)	0.5 (0.9)	0.8 (0.9)	0.7 (0.8)	0.143	0.1560
Upward SPV (°/s)	0.7 (0.6)	0.4 (0.6)	0.7 (0.5)	0.7 (0.6)	0.088	0.1173
Downward SPV (°/s)	0.7 (0.8)	0.3 (0.7)	0.7 (0.7)	0.8 (0.8)	**0.025**	**0.0375**

Bold values indicate statistically significant differences (*p* < 0.05). ^a^*p* < 0.05 vs. Mild group; ^b^*p* < 0.05 vs. Moderate group; ^c^*p* < 0.05 vs. Severe group.

Spearman’s analysis showed that right saccade accuracy, left saccade accuracy, right peak velocity, and left peak velocity were negatively correlated with total CSVD load (*p* < 0.05); right saccade latency and left saccade latency were positively correlated with total CSVD load (*p* < 0.05); and the obliquity angle and downward SPV values did not show a correlation with total CSVD load (see Table 3).

**Table 3:** Correlation analysis of eye movement performance and total load in CSVD.

Variables	*r*s	*p*	FDR-adjusted *P*-value
Right saccade accuracy (%)	−0.413	**<0.001**	**0.008**
Left saccade accuracy (%)	−0.438	**<0.001**	**0.004**
Right peak velocity (°/s)	−0.247	**0.007**	**0.009**
Left peak velocity (°/s)	−0.269	**0.003**	**0.005**
Right saccade latency (ms)	0.506	**<0.001**	**0.003**
Left saccade latency (ms)	0.396	**<0.001**	**0.002**
Obliquity angle (°)	0.106	0.250	0.250
Downward SPV (°/s)	0.146	0.111	0.127

Bold values indicate statistically significant differences (*p* < 0.05).

#### Logistic regression analysis of factors associated with increased total load in CSVD patients

Baseline analysis showed significant differences in age and hypertension among the three groups (*p* < 0.05). Ordinal logistic regression identified advancing age and a history of hypertension as independent risk factors for a higher total CSVD burden (*p* < 0.05). After adjusting for age and hypertension, right saccade accuracy, left saccade accuracy, right saccade latency, and left saccade latency were independently associated with increased total CSVD load. Left peak velocity showed a statistically significant association with total CSVD load (*p* = 0.049); however, the effect size was small (see Table 4).

**Table 4:** Multifactorial logistic regression analysis of risk factors for CSVD total load score.

Variables	*β*	S. E	Wald	*p*	OR	95%CI	
						Lower bound	Upper bound
Age	0.074	0.023	10.387	**0.001**	1.077	1.029	1.126
Hypertension	1.553	0.410	14.342	**<0.001**	4.726	2.115	10.560
Right saccade accuracy (%)	−0.080	0.019	17.080	**<0.001**	0.923	0.890	0.959
Left saccade accuracy (%)	−0.064	0.016	15.970	**<0.001**	0.938	0.908	0.968
Right peak velocity (°/s)	−0.006	0.003	3.761	0.052	0.994	0.988	1.000
Left peak velocity (°/s)	−0.006	0.003	3.862	0.049	0.994	0.988	1.000
Right saccade latency (ms)	0.015	0.004	14.788	**<0.001**	1.015	1.008	1.023
Left saccade latency (ms)	0.014	0.004	13.489	**<0.001**	1.014	1.007	1.022
Obliquity angle (°)	−0.864	0.615	1.976	0.160	0.422	0.126	1.406
Downward SPV (°/s)	−0.735	0.613	1.435	0.231	0.480	0.144	1.596

Bold values indicate statistically significant differences (*p* < 0.05).

**Figure 1:**
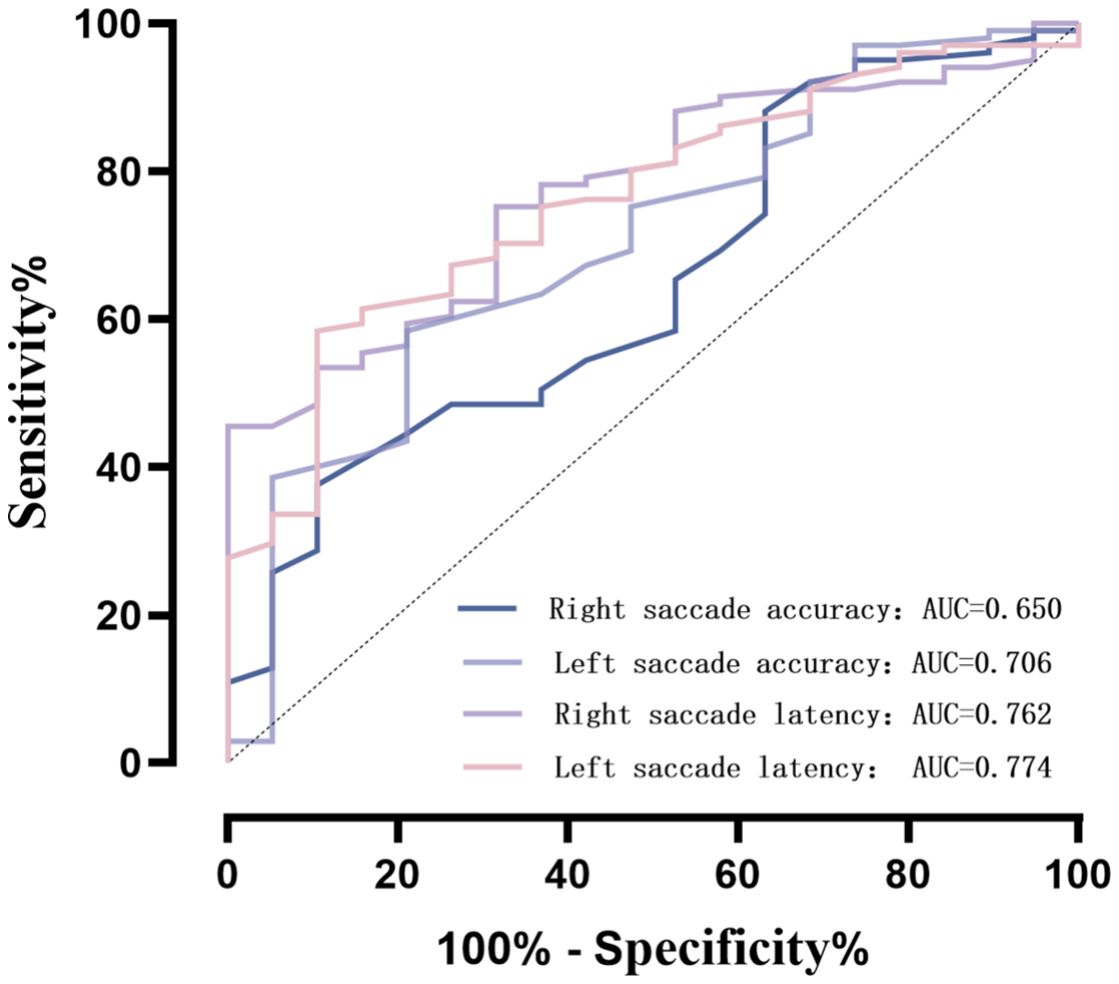
ROC curves of eye movements in identifying moderate-to-severe CSVD total loads.

The ROC analysis showed the discriminative ability of eye movement parameters for distinguishing moderate-to-severe CSVD burden. The AUC of right saccade accuracy and left saccade accuracy was 0.650 (95% CI 0.519–0.781, *p* = 0.039) and 0.706 (95% CI 0.580–0.831, *p* = 0.005), respectively. The optimal cut-off values were 70.5 and 78.5, with corresponding sensitivities of 89.5 and 78.9% and specificities of 37.6 and 58.4%, respectively. For saccade latency, the AUCs of right and left saccade latency were 0.762 (95% CI 0.658–0.866, *p* < 0.001) and 0.774 (95% CI 0.678–0.870, *p* < 0.001), respectively. The optimal cut-off values were 237 ms and 265.5 ms, with sensitivities of 58.4 and 45.5%, and specificities of 37.6 and 99.9%, respectively. Left peak velocity showed no significant discriminative ability (see Figure 1).

### Relationship between cognitive function and eye movement characteristics in CSVD patients

#### General data comparison between the normal cognitive function group and the cognitive impairment group

Among the 120 patients, 81 patients had complete cognitive function assessment data, including 45 patients in the normal cognitive function group and 36 patients in the cognitive impairment group. As detailed in Supplementary Table 1, no significant differences in baseline characteristics were observed between patients with complete data and those with missing data, indicating no substantial selection bias. The results showed that patients in the cognitive impairment group were older than those in the normal cognitive function group (*p* < 0.05). There were no differences in terms of gender, history of alcohol consumption, history of hypertension, history of diabetes mellitus, history of coronary heart disease, history of hyperlipidaemia, LDL and neutrophils (*p* > 0.05) (see Table 5).

**Table 5:** Comparison of general information between the normal cognitive function group and impaired cognitive function group.

Variables	Normal cognitive function group	Cognitive impairment group	Statistic (*t*/*x*^2^/*z*)	*p*
Total	45	36		
Age [M (IQR)]	67 (14)	71 (9)	−2.270	**0.023**
Sex			0.203	0.653
Female [*n* (%)]	19 (42.2)	17 (47.2)		
Male [*n* (%)]	26 (57.8)	19 (52.8)		
Alcohol [*n* (%)]	10 (22.2)	5 (13.9)	0.920	0.337
Hypertension [*n* (%)]	26 (57.8)	27 (75.0)	2.623	0.105
Diabetes [*n* (%)]	11 (24.4)	8 (22.2)	0.055	0.815
Coronary artery disease [*n* (%)]	6 (13.3)	5 (13.9)	0.005	0.942
Hyperlipidemia [*n* (%)]	9 (20.0)	9 (25.0)	0.289	0.591
LDL (mmol/l) [*x*^−^ ± *s*]	2.62 ± 0.86	2.59 ± 0.75	−0.43	0.667
Neutrophils (%) [*M* (IQR)]	64.1 (12.8)	63.7 (8.8)	−0.854	0.393
MoCA [*x*^−^ ± *s*]	24.2 ± 3.94	15.5 ± 5.5	8.314	**<0.001**

Bold values indicate statistically significant differences (*p* < 0.05).

The mean MoCA score was significantly lower in the cognitive impairment group than in the normal cognition group (*p* < 0.001). Detailed comparisons of individual MoCA cognitive domains are presented in Supplementary Table 2. Among patients with cognitive impairment, moderate cognitive impairment was the most common subtype (58.3%), followed by mild (25.0%) and severe cognitive impairment (16.7%) (see Supplementary Table 3).

#### Eye movement characteristics of patients in the normal cognitive function group and the cognitive impairment group

The patients in the 2 groups differed in right saccade accuracy, left saccade accuracy, right peak velocity, left peak velocity in the saccading experiment (*p* < 0.05). There were no differences in right saccade latency, left saccade latency in the saccading experiment, obliquity angle in the alternating masking experiment, and centered SPV value, rightward SPV, leftward SPV value, upward SPV value, and downward SPV value in the gaze experiment (*p* > 0.05) (see Table 6).

**Table 6:** Comparison of eye movement data between the normal cognitive function group and the impaired cognitive function group.

Variables	Normal cognitive function group	Cognitive impairment group	Statistic (*t*/*x*^2^/*z*)	*p*	FDR-adjusted *P*-value
Right saccade accuracy (%)	77.07 ± 11.42	68.14 ± 10.40	3.636	**<0.001**	**0.0120**
Left saccade accuracy (%)	79.56 ± 13.70	70.61 ± 12.14	3.070	**0.003**	**0.0180**
Right peak velocity (°/s)	271.04 ± 64.96	239.47 ± 44.83	2.480	**0.015**	**0.0450**
Left peak velocity (°/s)	289.00 ± 55.69	253.14 ± 61.43	2.751	**0.007**	**0.0280**
Right saccade latency (ms)	238.73 ± 57.39	256.78 ± 53.34	−1.451	0.151	0.3020
Left saccade latency (ms)	258.69 ± 49.30	267 ± 61.80	−0.674	0.503	0.6707
Obliquity angle (°)	0.9 (1)	0.9 (1)	−0.457	0.647	0.7058
Centered SPV (°/s)	0.6 (0.6)	0.5 (0.6)	−1.321	0.186	0.3189
Leftward SPV (°/s)	0.6 (0.6)	0.6 (0.8)	−0.591	0.555	0.6660
Rightward SPV (°/s)	0.5 (0.6)	0.8 (1.3)	−2.241	**0.025**	**0.0600**
Upward SPV (°/s)	0.6 (0.6)	0.6 (0.6)	−0.029	0.977	0.9770
Downward SPV (°/s)	0.7 (0.6)	0.8 (0.8)	−1.125	0.261	0.3915

Bold values indicate statistically significant differences (*p* < 0.05).

To further evaluate the relationship between eye movement parameters and cognitive performance, Spearman correlation analyses were performed using continuous MoCA scores. Lower saccade accuracy and peak velocity, together with prolonged right saccade latency, were significantly associated with lower MoCA scores. These associations remained statistically significant after false discovery rate (FDR) correction (see Supplementary Table 4).

#### Logistic regression analysis of factors influencing cognitive function in patients with CSVD

Baseline analysis showed significant age differences between the cognitive and non-cognitive impairment groups (*p* < 0.05). To control for potential confounders, binary logistic regression was performed adjusting for age, sex, hypertension, diabetes mellitus, smoking and drinking history, and years of education. Following full covariate adjustment, advancing age was an independent risk factor for cognitive impairment in CSVD patients (*p* < 0.05). Additionally, bilateral saccade accuracy, bilateral peak velocity, and rightward SPV were independently associated with cognitive impairment (*p* < 0.05) (see Table 7).

**Table 7:** Logistic regression analysis of risk factors for cognitive function in CSVD patients.

Variables	*β*	S. E	Wald	*p*	OR	95%CI	
						Lower bound	Upper bound
Age	0.084	0.034	6.054	**0.014**	1.088	1.018	1.163
Right saccade accuracy (%)	−0.080	0.027	8.379	**0.004**	0.924	0.876	0.973
Left saccade accuracy (%)	−0.057	0.023	6.342	**0.012**	0.944	0.903	0.988
Right peak velocity (°/s)	−0.012	0.005	4.685	**0.030**	0.988	0.978	0.998
Left peak velocity (°/s)	−0.014	0.005	7.184	**0.007**	0.986	0.976	0.996
Rightward SPV (°/s)	1.541	0.525	8.603	**0.003**	4.668	1.669	13.066

Bold values indicate statistically significant differences (*p* < 0.05).

**Figure 2:**
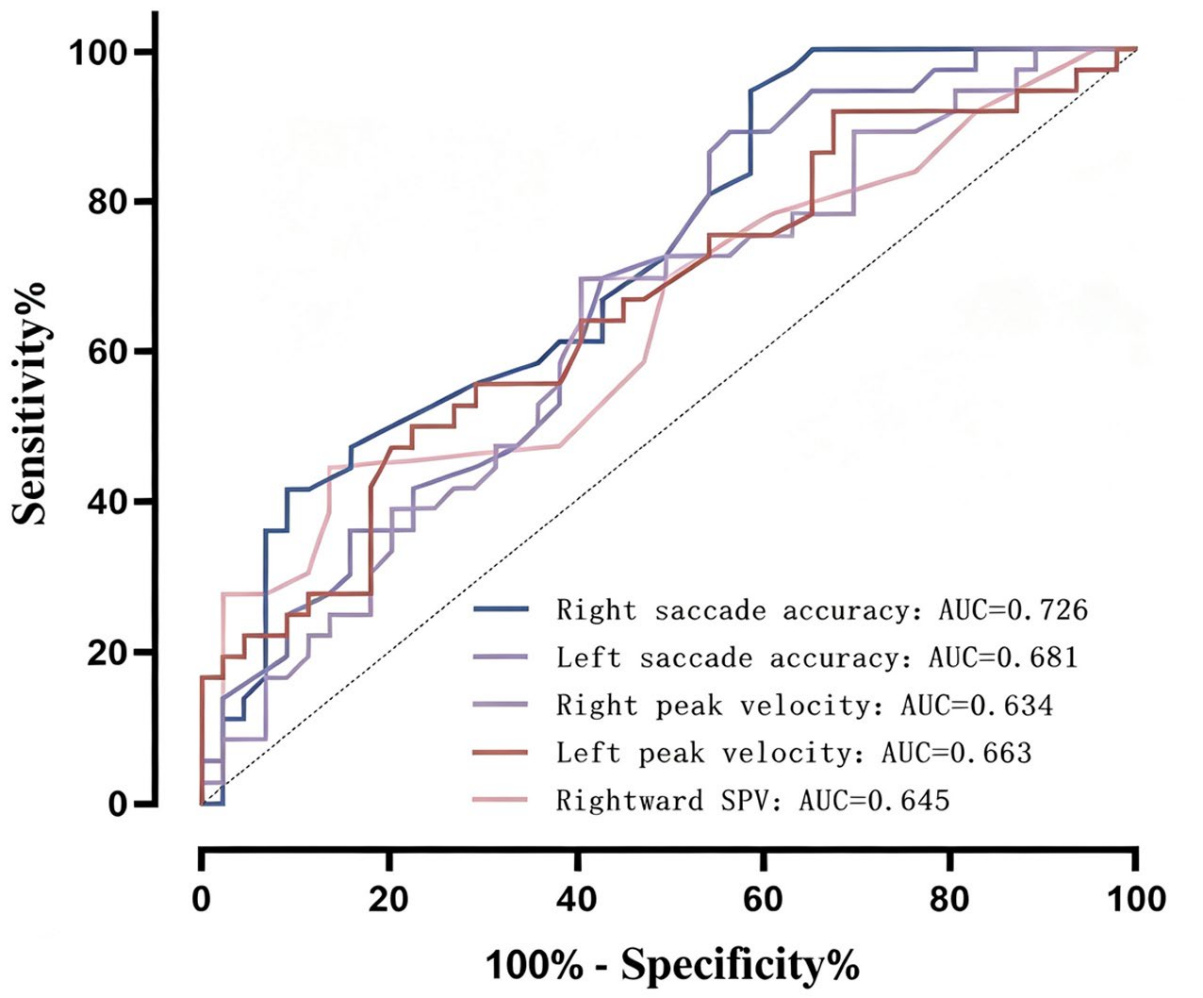
ROC curves of eye movements in identifying cognitive impairment.

The ROC analysis demonstrated the discriminative performance of eye movement parameters for identifying cognitive impairment in patients with CSVD. The AUCs for right and left saccade accuracy were 0.726 (95% CI 0.617–0.835, *p* = 0.001) and 0.681 (95% CI 0.567–0.796, *p* = 0.005), respectively. The optimal cut-off values were 80.5 and 82.5, with sensitivities of 42.2 and 44.4%, and specificities of 94.4 and 88.9%, respectively. For saccade peak velocity, the AUCs were 0.634 (95% CI 0.513–0.755, *p* = 0.040) and 0.663 (95% CI 0.543–0.783, *p* = 0.012) for right and left eyes, respectively. The optimal cut-off values were 248 and 243.5, with sensitivities of 60.0 and 77.8%, and specificities of 69.4 and 77.8%, respectively. Rightward SPV showed an AUC of 0.645 (95% CI 0.523–0.768, *p* = 0.026), with sensitivity 44.4% and specificity 86.7% (see Figure 2).

## Discussion

Cerebral small vessel disease (CSVD) is a major risk factor for neurological injury in the elderly (18), and its early clinical manifestations are insidious and can only be diagnosed through imaging, so exploring the characteristics of CSVD is urgent. This study investigated the associations between quantitative oculomotor characteristics, MRI-defined CSVD burden, and cognitive function. We found that while reduced saccade accuracy was a common feature associated with both higher MRI burden and cognitive impairment, other oculomotor parameters exhibited distinct association patterns. Specifically, prolonged saccade latency was independently associated with the structural burden of CSVD, whereas reduced peak velocity was more closely linked to cognitive impairment. These findings suggest that different oculomotor parameters reflect distinct functional manifestations of CSVD rather than representing a uniform consequence of small vessel injury.

The divergent associations of latency and peak velocity offer insight into the underlying pathophysiology. The independent association between prolonged latency and MRI burden suggests that the cumulative structural lesion load, particularly diffuse white matter injury, may preferentially disrupt the efficiency of neural transmission within long-range pathways linking frontal, parietal, and brainstem regions (19). This structural disconnection likely slows the relay of information required for saccade initiation. In contrast, the association between reduced peak velocity and cognitive impairment, independent of MRI burden, implies that peak velocity may better reflect the functional integrity of cortical–subcortical networks supporting executive control and attention (20). This aligns with the concept that cognitive impairment in CSVD arises more from network disruption than from the simple volume of ischemic lesions (21–26).

Our findings extend previous research by concurrently analyzing total CSVD burden, cognitive status, and detailed oculomotor metrics. While prior studies have linked eye movement abnormalities to cognitive decline in neurodegenerative diseases, few have dissected these relationships within the context of quantified CSVD burden. Existing literature highlights various oculomotor deficits across different conditions: Alichniewicz observed impaired anti-saccade performance in mild cognitive impairment but did not assess saccade accuracy or velocity (27); Pavišić reported increased spontaneous eye movements, reduced accuracy, and prolonged latency in dementia (28); and Vintonyak identified distinct oculomotor signatures in other degenerative diseases, such as increased saccadic frequency in Parkinson’s disease, saccadic intrusions during smooth pursuit in multiple system atrophy, and slowed vertical saccades in progressive supranuclear palsy (29). Furthermore, increased gaze variability has been documented in Alzheimer’s disease (30). In the specific context of CSVD, Pinkhardt suggested that eye movement abnormalities are primarily driven by cognitive deterioration rather than white matter lesion burden (31). Consistent with this, our observation that cognitive impairment is associated with a specific oculomotor profile (reduced accuracy and peak velocity) that only partially overlaps with the MRI-defined structural burden reinforces the concept that functional network impairment can exist independently of gross anatomical damage (32). Furthermore, the absence of significant vestibular abnormalities, such as spontaneous nystagmus, in our cohort is an informative negative finding. It suggests that chronic CSVD preferentially targets the supranuclear networks responsible for voluntary saccadic control, while largely sparing the reflexive brainstem pathways governing the vestibulo-ocular reflex.

From a clinical perspective, VNG offers a promising, objective tool to complement existing diagnostic methods for CSVD. Unlike many cognitive screening tests, VNG is largely unaffected by a patient’s language proficiency or educational background, providing a more universal measure of brain function (33). Although individual parameters may have modest effect sizes and are not suitable as standalone diagnostic tests, a comprehensive analysis of multiple saccadic indices can provide a richer, more nuanced picture of a patient’s neurological status. The distinct patterns we observed—where latency may serve as a proxy for structural burden and peak velocity for cognitive functional capacity—highlight the value of a multidimensional approach. This functional assessment could be particularly useful for monitoring disease progression or response to therapy in a way that structural imaging alone cannot.

The primary strength of this study is the simultaneous evaluation of quantitative eye movements, validated total CSVD burden scores, and cognitive performance within a single, well-characterized cohort. This design allowed for a direct comparison of how structural and functional disease manifestations relate to specific oculomotor deficits. The use of the total CSVD burden score, which integrates multiple MRI markers, provides a more holistic measure of small vessel injury than assessing markers in isolation. Our findings were also robust, confirmed using both categorical (cognitive impairment vs. normal) and continuous (MoCA score) measures of cognitive function, with appropriate corrections for multiple comparisons.

Several limitations warrant consideration. First, the cross-sectional design prevents us from drawing conclusions about causality or the temporal sequence of these changes. It remains to be seen whether worsening oculomotor performance predicts future cognitive decline or simply co-occurs with it. Second, as participants were recruited from a single tertiary care center, the cohort may be biased toward more severe CSVD cases, limiting the generalizability of our findings to milder or community-dwelling populations. The lack of a healthy control group also means we cannot determine if the observed abnormalities are specific to CSVD or represent an exaggeration of normal aging. Finally, our analysis was limited to conventional VNG parameters. Future studies incorporating more complex tasks, smooth pursuit analysis, and advanced neuroimaging like diffusion tensor imaging (DTI) could provide a more detailed map of the specific neural circuits underlying these oculomotor deficits.

In conclusion, quantitative oculomotor abnormalities are independently associated with both the structural burden of CSVD and the severity of cognitive impairment, but they illuminate different facets of the disease. Reduced saccade accuracy emerges as a consistent marker of general network dysfunction, while prolonged saccade latency and reduced peak velocity appear to be more specific indicators of structural lesion burden and cognitive functional impairment, respectively. These findings support the utility of quantitative eye movement assessment as a valuable, non-invasive tool for probing cerebral network integrity in patients with CSVD.

## Data Availability

The original contributions presented in the study are included in the article/Supplementary material, further inquiries can be directed to the corresponding author.
