# Supplementary material for: Eye movement characteristics and their correlation with cognitive function in cerebral small vessel disease
Source: Front Neurol. 2026 Aug 11;17:1878897. doi: 10.3389/fneur.2026.1878897 (PMC13503216; doi:10.3389/fneur.2026.1878897)
Supplement: Supplementary file 1 [file Table_1.DOCX]

Supplementary Table 1 Comparison of baseline characteristics between patients with complete and missing cognitive data.

|  | Complete cognitive data  (n=81) | Missing cognitive data  (n=39) | P |
| --- | --- | --- | --- |
| Age,years  [M (IQR)] | 68 (12) | 65 (15) | 0.176 |
| Sex |  |  | 0.535 |
| Female [n (%)] | 36 (44.4) | 15 (38.5) |  |
| Male [n (%)] | 45 (55.6) | 24 (61.5) |  |
| Smoking [n (%)] | 23 (28.4) | 17 (43.6) | 0.098 |
| Alcohol [n (%)] | 15 (18.5) | 9 (23.1) | 0.559 |
| Hypertension  [n (%)] | 53 (65.4) | 29 (74.4) | 0.325 |
| Diabetes [n (%)] | 19 (23.5) | 5 (12.8) | 0.172 |
| Coronary artery disease [n (%)] | 11 (13.6) | 6 (15.4) | 0.791 |
| LDL (mmol/l)  [M (IQR)] | 2.54 (1.13) | 2.72 (1.61) | 0.944 |
| Neutrophils (%) | 63.7 (12.6) | 60.1 (16.2) | 0.471 |
| [M (IQR)] |  |  |  |

Supplementary Table 2. Comparison of MoCA cognitive domain scores between participants with normal cognition and cognitive impairment.

|  | Normal cognition | Cognitive impairment |
| --- | --- | --- |
| Visuospatial/  Executive function | 4.20±1.32 | 2.64±1.62 |
| Naming | 2.91±0.36 | 2.42±0.94 |
| Attention | 5.18±1.17 | 3.25±1.48 |
| Language | 2.31±0.87 | 1.11±0.75 |
| Abstraction | 1.56±0.62 | 0.89±0.62 |
| Delayed recall | 1.87±1.01 | 0.81±1.09 |
| Orientation | 5.87±0.40 | 4.67±1.80 |

Supplementary Table 3. Distribution of cognitive impairment severity according to MoCA scores.

| Impairment grade | Cases, n (%) |
| --- | --- |
| Mild cognitive impairment | 9 (25.0) |
| Moderate cognitive impairment | 21 (58.3) |
| Severe cognitive impairment | 6 (16.7) |
| Total | 36 (100.0) |

### Supplementary Table 4. Spearman correlation analysis between eye movement parameters and continuous MoCA scores.

|  | rs | *p* | *FDR-adjusted*  *P value* |
| --- | --- | --- | --- |
| Right saccade accuracy (%) | -0.275 | **0.013** | **0.039** |
| Left saccade accuracy (%) | -0.337 | **0.002** | **0.024** |
| Right peak velocity (°/s) | -0.300 | **0.006** | **0.036** |
| Left peak velocity (°/s) | -0.297 | **0.007** | **0.028** |
| Right saccade latency (ms) | 0.255 | **0.021** | **0.050** |
| Left saccade latency (ms) | 0.143 | 0.201 | 0.402 |
| Obliquity angle (°) | 0.083 | 0.457 | 0.548 |
| Centered SPV (°/s) | 0.094 | 0.401 | 0.535 |
| Left SPV (°/s) | 0.073 | 0.516 | 0.563 |
| Right SPV (°/s) | 0.121 | 0.283 | 0.485 |
| Superior SPV (°/s) | 0.030 | 0.791 | 0.791 |
| Downward SPV (°/s) | 0.113 | 0.314 | 0.471 |
